# The lactylation-immunosuppression network in cancer: driving a metabolic-epigenetic axis

**DOI:** 10.3389/fimmu.2026.1752934

**Published:** 2026-01-26

**Authors:** Jinfeng Ye, Yunliang Lu, Wansu Huang, Shan Huang, Zhe Zhang, Xiaoying Zhou, Xue Xiao, Tingting Huang

**Affiliations:** 1 Department of Otorhinolaryngology-Head and Neck Surgery, First Affiliated Hospital of Guangxi Medical University, Nanning, China; 2 Medical Simulator Center, First Affiliated Hospital of Guangxi Medical University, Nanning, China; 3 Department of Clinical Laboratory, First Affiliated Hospital of Guangxi Medical University, Nanning, China; 4 Key Laboratory of Clinical Laboratory Medicine of Guangxi Department of Education, Nanning, China; 5 Guangxi Key Laboratory of Early Prevention and Treatment for Regional High Frequency Tumor, Guangxi Medical University, Nanning, China; 6 Life Science Institute, Guangxi Medical University, Nanning, China; 7 Department of Radiation Oncology, First Affiliated Hospital of Guangxi Medical University, Nanning, China

**Keywords:** immune escape, immunosuppression, lactylation, metabolic reprogramming, tumor microenvironment

## Abstract

The accumulation of lactate in the tumor microenvironment (TME), driven by the Warburg effect, is closely associated with immunosuppression. Lactate can contribute to this process through lysine lactylation, a novel post-translational modification. We propose a conceptual framework, the “Lactylation-immunosuppression network,” that links tumor metabolic reprogramming to immune cell signaling and gene expression. This network highlights a metabolic-epigenetic axis linking lactylation to immunosuppression via a synergistic dual mechanism: long-term epigenetic programming via histone lactylation establishes a stable immunosuppressive transcriptome, while rapid, dynamic non-histone lactylation directly modulates protein activity and stability, thereby potentiating function. This review summarizes how lactylation may undermine anti-tumor immunity by remodeling myeloid and T cell compartments, fortifying immune checkpoint barriers, and creating self-reinforcing metabolic feedback loops. By elucidating this mechanism, we highlight novel therapeutic targets, propose a “kinetic threshold” model to resolve the paradoxical role of lactate, and provide a unified conceptual framework for developing next-generation immunotherapies and guiding future mechanistic studies.

## 1 Introduction

The tumor microenvironment (TME) is characterized by severe metabolic disorders (1, 2). To promote rapid proliferation, cancer cells adopt the Warburg effect – preferential aerobic glycolysis, which leads to the accumulation of a large amount of lactate (1, 3). For a long time, lactate has been regarded as a simple metabolic waste product. Still, it is now increasingly recognized as a metabolite and signaling cue implicated in an immunosuppressive program (4, 5). In fact, the continuous accumulation of metabolic by-products in the TME can regulate the function and signaling components of immune cells, positioning lactate as a central node in tumor immunometabolism. In 2019, a landmark study revealed the deeper function of lactate: it drives a new post-translational modification, lysine lactylation (6).

**Figure 1:**
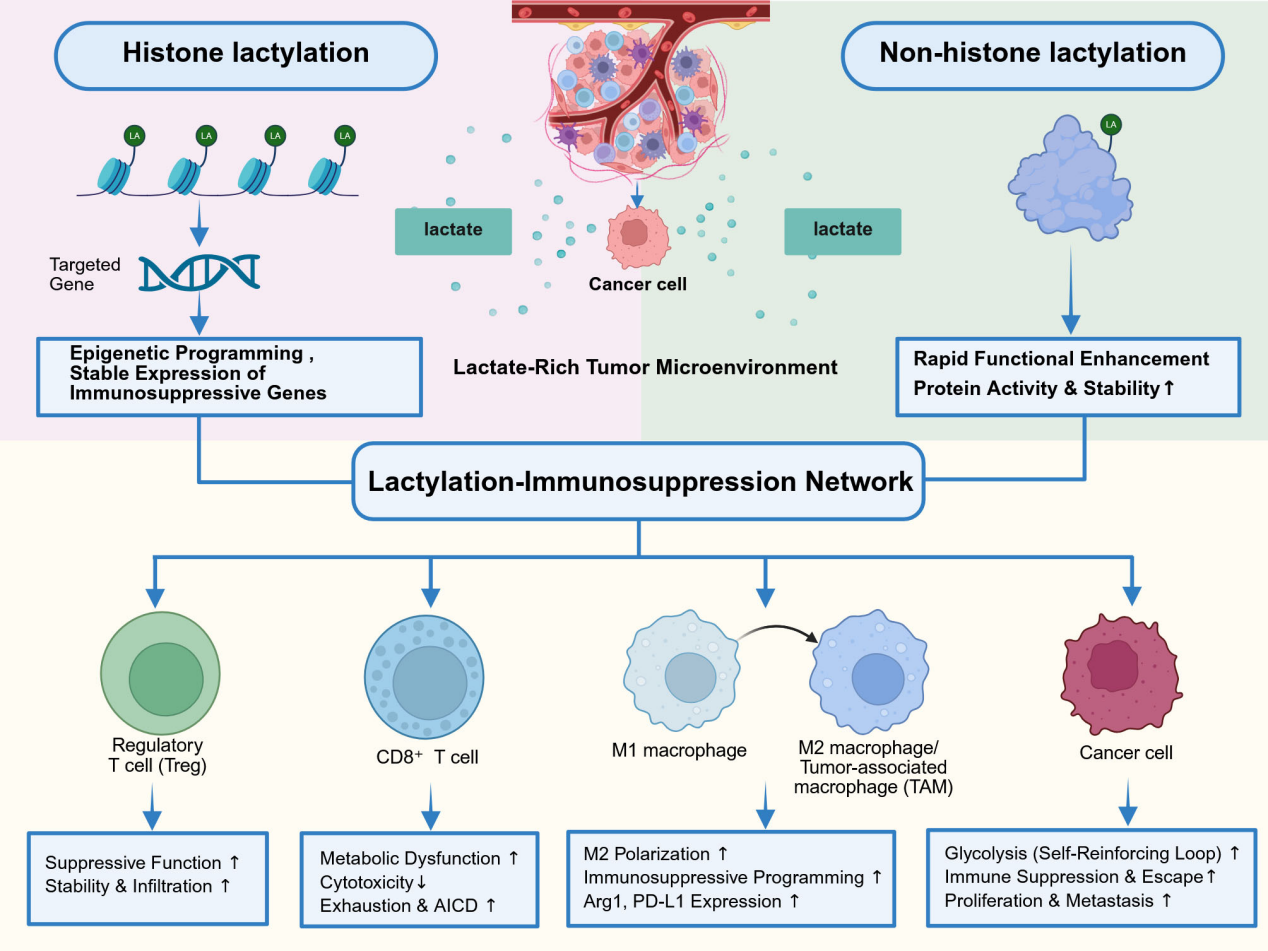
The lactylation-immunosuppression network: a synergistic metabolic-epigenetic axis in the tumor microenvironment. This figure illustrates the conceptual framework of the Lactylation-Immunosuppression Network, a dual-regulatory network associated with the lactate-rich tumor microenvironment. Cancer cells, via the Warburg effect, secrete excess lactate to engage two distinct signaling arms: (Left) Histone lactylation functions as a durable epigenetic mechanism, driving the stable expression of immunosuppressive genes; (Right) Non-histone lactylation acts as a rapid functional switch, modulating protein activity and stability. These pathways synergize to systematically remodel the immune network (1): Potentiating the suppressive function and stability of Regulatory T cells (Tregs) (2); Inducing metabolic dysfunction and exhaustion in CD8^+^ T cells (3); Promoting M2 polarization in macrophages via immunosuppressive programming; and (4) Establishing a glycolytic self-reinforcing loop within cancer cells that reinforces immune evasion and metastasis. Created with BioRender.com.

If these modifications are considered in isolation, they still cannot fully explain tumor immunosuppression. We therefore propose that they operate as a synergistic regulatory architecture – the “Lactylation-immunosuppression network,” which we use here as a conceptual metabolic-epigenetic axis. In this model, histone lactylation can contribute to a long-term epigenetic program for immunosuppressive genes. In contrast, non-histone lactylation tends to potentiate function by directly altering protein activity and stability rapidly. This review first deconstructs the network’s architectural patterns of synergy in key immune cells, then examines the metabolic engine of self-reinforcing feedback loops that sustain it, discusses emerging therapeutic strategies aimed at targeting key nodes to dismantle it, and finally defines the critical challenges and future perspectives that will shape the next decade of research in this field, providing a new conceptual framework for cancer therapy (**Figure 1**).

## 2 Deconstructing the network: a synergistic system of immune suppression

The Lactylation-Immunosuppression Network can be conceptualized as a coordinated, dual-regulatory mechanism in which long-term epigenetic programming by histone lactylation is functionally enhanced by rapid, dynamic modulation by non-histone lactylation. This adaptive synergy tends to weaken the anti-tumor immune response, as described in **Table 1**. For example, myeloid cells exhibit “sequential synergy,” where earlier events set the stage for later ones; T cells, on the other hand, demonstrate “parallel enhancement,” with multiple independent processes converging. This functional difference provides essential evidence for identifying therapeutic targets corresponding to different cell subsets.

**Table 1:** Key histone and non-histone targets linked to the lactylation-immunosuppression network.

Target protein	Specific site	Cell type	Key function	References
Histone H3	K18	Macrophages (TAMs)	Activates M2-polarizing genes (*ARG1*, *VEGF*), immunosuppressive cytokines (*IL-10*, *TGF-β*), metabolic regulator (*GPD2*) and checkpoints (*PD-L1*, *SIRPA*) to drive immunosuppressive polarization.	(8, 10–13)
Histone H3	K18	TIMs	Activates transcription of the METTL3 gene.	(17)
Histone H3	K9, K18	CD8^+^ T cells (Physiological state)	Maintains mitochondrial homeostasis for normal T cell function.	(24)
Histone H3	K18	CD8^+^ T cells (High-lactate TME)	Upregulates inhibitory factors (ANGPTL4, circATXN7) to program T cell dysfunction and sensitivity to AICD.	(28–30)
Histone H3	K18	Tregs	Enhances Treg immunosuppressive machinery (CCR8, CD39/73, TNFR2).	(31, 32)
Histone H3	K14, K18	Tumor cells	Transcriptionally activates PD-L1 expression for immune evasion.	(35–37)
Histone H3	K18	Tumor cells	Upregulates immune checkpoints (B7-H3, CD47) to suppress T cells and evade phagocytosis.	(38, 39)
Histone H3	K9, K18	TME communication network	Activates expression of pro-tumoral factors, including inhibitory cytokines (IL-10, IL-11), chemokines (CXCL1, CXCL5, CCL18), and adhesion molecules (VCAM1).	(42–46)
Histone H3	K18	Tumor cells	Promotes glycolysis by stabilizing PI3K/AKT/HIF-1α signaling via USP39.	(47)
Histone H3	K18	Tumor cells	Drives Warburg effect by upregulating glycolytic enzymes (via SRSF10) and suppressing OXPHOS (via ACAT2).	(48, 49)
Histone H3	K18	Tumor cells	Activates expression of the autophagy enhancer protein RUBCNL.	(55)
PKM2	K62	Macrophages	Enhances pyruvate kinase activity to provide metabolic support for M2 polarization.	(14)
RIG-I	K852	Macrophages	Inhibits its ability to activate NF-κB signaling, promoting M2 macrophage polarization.	(15)
METTL3	K281, K345	TIMs	Enhances m6A writer activity to sustain pro-tumor JAK-STAT3 signaling.	(17)
MOESIN	K72	Tregs	Amplifies TGF-β signaling to enhance Treg suppressive function	(33)
APOC2	K70	Tregs	Enhances extracellular lipolysis and FFA release to support Treg survival and expansion.	(34)
PD-L1	K270	Tumor cells	Inhibits degradation, increasing protein stability and surface expression for potent CD8^+^ T cells suppression.	(40, 41)
HIF1α	unspecified	Tumor cells	Enhances protein stability, enabling sustained transcriptional activation of downstream glycolytic genes.	(50)
NUSAP1	K34	Tumor cells	Stabilizes protein to drive glycolysis-lactate feedback loop via LDHA.	(51)
eEF1A2	K408	Tumor cells	Promotes protein synthesis to support tumor proliferation.	(53)
SOX9	unspecified	Tumor cells	Maintains cancer stemness, migration, and invasion.	(54)
Vps34	K356, K781	Tumor cells	Enhances kinase activity to promote the autophagy process.	(56)

ACAT2, Acetyl-CoA Acetyltransferase 2; AICD, activation-induced cell death; ANGPTL4, Angiopoietin-like 4; APOC2, Apolipoprotein C-II; Arg1, Arginase 1; B7-H3, B7 Homolog 3; CCL18, C-C Motif Chemokine Ligand 18; CCR8, C-C Motif Chemokine Receptor 8; circATXN7, Circular RNA ATXN7; CXCL: C-X-C Motif Chemokine Ligand; eEF1A2, Eukaryotic Translation Elongation Factor 1 Alpha 2; FFA, Free Fatty Acids; GPD2, Glycerol-3-Phosphate Dehydrogenase 2; HIF-1α, Hypoxia-inducible Factor 1-alpha; JAK, Janus Kinase; LDHA, Lactate Dehydrogenase A; METTL3, Methyltransferase-like 3; MOESIN, Membrane-Organizing Extension Spike Protein; NF-κB, Nuclear factor kappa-light-chain-enhancer of activated B cells; NUSAP1, Nucleolar and spindle associated protein 1; OXPHOS, Oxidative phosphorylation; PD-L1, Programmed death-ligand 1; PFKP, Phosphofructokinase, platelet; PI3K, Phosphoinositide 3-kinase; PKM2, Pyruvate kinase M2; RIG-I, Retinoic acid-inducible gene I; RUBCNL, Rubicon-like autophagy enhancer; SIRPα, Signal regulatory protein alpha; SOX9, SRY-Box Transcription Factor 9; SRSF10, Serine and Arginine Rich Splicing Factor 10; STAT3, Signal transducer and activator of transcription 3; TAMs, tumor-associated macrophages; TGF-β, Transforming growth factor beta; TIMs, tumor-infiltrating myeloid cells; TNFR2, Tumor necrosis factor receptor 2; Treg, Regulatory T cell; USP39, Ubiquitin Specific Peptidase 39; VCAM1, Vascular cell adhesion molecule 1; Vegf, Vascular endothelial growth factor; Vps34, Vacuolar protein sorting 34.

Myeloid compartment (tumor-associated macrophages, TAMs): In myeloid cells, particularly TAMs, the Lactylation-Immunosuppression Network appears to drive pro-tumor M2 polarization (7–9). Histone lactylation (especially Histone H3 Lysine 18 lactylation (H3K18la)) is enriched at promoters of M2-polarizing genes [e.g., Arginase 1 (ARG1), Vascular Endothelial Growth Factor (VEGFA) (10)], immunosuppressive cytokine genes [e.g., Interleukin-10 (IL-10), Transforming Growth Factor-beta (TGF-β) (8)], and immune checkpoint genes [Programmed Death-Ligand 1 (PD-L1, CD274) and Signal Regulatory Protein Alpha (SIRPA) (11)]. This pro-tumoral signature is maintained by regulating metabolism [e.g., Glycerol-3-Phosphate Dehydrogenase 2 (GPD2) (12)] and by activating key signaling cascades, such as those involving TNF Receptor Associated Factor 6 (13). This foundational process is then functionally potentiated by non-histone lactylation of metabolic and signaling proteins. For instance, lactylation of Retinoic Acid-Inducible Gene I (an innate immune sensor) inhibits its ability to activate Nuclear Factor kappa-light-chain-enhancer of activated B cells (NF-κB) signaling, potentially forming feed-forward loops (15, 16). A typical example is the two-step activation of Methyltransferase-like 3 (METTL3) in tumor-infiltrating myeloid cells. H3K18la controls METTL3 expression. Lactylation of METTL3 was shown to boost its enzymatic activity and thus helps maintain the immunosuppressive Janus Kinase (JAK)/Signal Transducer and Activator of Transcription 3 (STAT3) signaling (17).

Lymphoid compartment (T Cells): Evidence suggests the Lactylation-Immunosuppression Network can weaken T cells’ anti-tumor response. It does this in two ways: it inhibits CD8^+^ T cells while simultaneously enhancing regulatory T cells (Tregs). High levels of tumor lactylation result in fewer CD8^+^ T cells entering the tumor and a greater number of Tregs, an imbalance that disrupts antitumor immunity (18–20). This synergistic action is consistent with the parallel reinforcement of the immunosuppressive state. This method differs from the sequential type of amplification, for instance the METTL3 cascade, which is observed in myeloid cells.

For CD8^+^ T cells, the central role of lactylation is functional inhibition, and its effect is context-dependent. Under normal activation, CD8^+^ T cells shift from oxidative phosphorylation (OXPHOS) to glycolysis for energy (21–23), and basal intracellular lactate could support H3K18la and Histone H3 Lysine 9 lactylation (H3K9la) to fine-tune mitochondrial homeostasis. However, the high-lactate TME subverts this adaptation (24). By blocking lactate efflux from CD8^+^ T cells, tumor-derived lactate can cause intracellular acidification and an imbalance in NAD^+^/NADH, weakening T-cell cytotoxicity (25). In addition to direct inhibition, lactylation also supports surviving CD8^+^ T cells becoming lactate consumers by upregulating lactate dehydrogenase B (*LDHB*) and mitochondrial pyruvate carrier (*MPC*) to maintain the tricarboxylic acid cycle (26, 27). This metabolic adaptation is a typical feature of exhausted T cells (26). Finally, histone lactylation may help stabilize this damaged state through epigenetic mechanisms by upregulating inhibitory factors [e.g., Angiopoietin-like 4 (*ANGPTL4*)] and increasing sensitivity to activation-induced cell death (via Circular RNA *ATXN7*), so that CD8^+^ T cells remain in a long-term state of exhaustion, forming a cell population that is prone to dysfunction (28–30).

For Tregs, however, lactylation enhances function and survival. Histone lactylation was shown to shape an immunosuppressive program: H3K18la upregulates C-C Motif Chemokine Receptor 8 (*CCR8*) (promotes Tregs migration to tumors) and Ectonucleoside Triphosphate Diphosphohydrolase-1 (*ENTPD1*)/Ecto-5’-nucleotidase (*NT5E*) (encoding enzymes CD39 and CD73 to generate immunosuppressive adenosine) (31). It also increases Tumor Necrosis Factor Receptor 2 (*TNFRSF1B*) expression, enhancing Tregs stability (32). CCR8^+^ Tregs also accumulate in lactate-rich TMEs (16), further strengthening immunosuppression. On the other hand, non-histone lactylation has been shown to provide additional function and metabolic support: Membrane-Organizing Extension Spike Protein (MOESIN) lactylation enhances TGF-β receptor signaling (33), which promotes immunoregulation and inhibits CD8^+^ T cell activity (16). In Tregs, lactylation of apolipoprotein C-II promotes lipolysis to produce fatty acids, the nutrients crucial for Tregs growth in the TME (34). This may contribute to lower effector T-cell activity and promote tumor tolerance.

Lactylation appears to further strengthen the immune regulatory network by upregulating immune checkpoints and remodeling intercellular signaling. Histone lactylation, especially H3K18la and Histone H3 Lysine 14 lactylation (H3K14la), promotes the transcription of checkpoint molecules such as CD274, B7 Homolog 3 (B7-H3), and Cluster of Differentiation 47 (CD47) (35–39). At the same time, non-histone lactylation of PD-L1 might occur, blocking its ubiquitinization and degradation, and enhancing its stability and membrane expression (40, 41). Lactylation can also alter the signaling of cytokines and chemokines. Marks like H3K9la and H3K18la are associated with increased transcription of inhibitory cytokines [e.g., *IL-10*, Interleukin-11 (*IL-11*)] and chemokines [e.g., C-X-C Motif Chemokine Ligand 1 (*CXCL1*), *CXCL5* and C-C Motif Chemokine Ligand 18 (*CCL18*)] (42–45); these factors recruit inhibitory myeloid cells. These signal molecules and adhesion molecules such as Vascular Cell Adhesion Molecule 1, work together to support immune escape (46). The lactylation of key signal intermediates could further enhance immunosuppression. Lactylation-driven METTL3 activation in myeloid cells can amplify downstream STAT3 signaling, potentially forming a malignant feedback loop maintained by IL-10 and Interleukin-6 (IL-6) (17). In aggregate, the Lactylation-Immunosuppression Network links metabolic cues (lactate) to immune signaling pathways at every level: epigenetic programming, modulation of signal transducers, and alteration of extrinsic cytokine-checkpoint milieus. Through parallel suppression of effector immunity and promotion of regulatory axes, this network is well placed to help sustain a highly resilient state of immune escape (16, 17).

## 3 Reinforcing the network: a self-regulating metabolic engine

The tumor may reinforce its Lactylation-Immunosuppression Network through a powerful self-reinforcing loop. This glycolysis-lactate-lactylation loop could couple malignant progression with a continuous lactate supply for the immunosuppressive TME.

First, histone lactylation can epigenetically program an amplified glycolytic state by upregulating key enzymes and factors. In endometrial cancer, H3K18la upregulates the deubiquitinase ubiquitin-specific peptidase 39 (USP39), thereby stabilizing the phosphoinositide 3-kinase/protein kinase B/hypoxia-inducible factor 1-alpha (HIF-1α) signaling pathway (47). In liver cancer, H3K18la increases expression of the RNA-binding protein Serine/arginine-rich splicing factor 10 (*SRSF10*), which upregulates multiple glycolytic proteins [phosphoglycerate kinase 1 (*PGK1*), glucose transporter 1 (GLUT1, *SLC2A1*), and lactate dehydrogenase A (*LDHA*)] (48). Additionally, histone lactylation can inhibit OXPHOS to enforce reliance on glycolysis: H3K18la induces Acetoacetyl-CoA Thiolase 2 (ACAT2), which acetylates and stabilizes Mitochondrial carrier homolog 2, impairing mitochondrial electron transport and pushing cells into a glycolytic overdrive (49).

Second, non-histone lactylation can functionally enhance the glycolytic program. Lactylation of key transcription factors and metabolic enzymes increases their stability or activity, further boosting glycolytic flux. For example, lactylation stabilizes HIF-1α, prolonging its activity and sustaining the expression of glycolysis-related genes (50). Similarly, in pancreatic cancer, lactylation of microtubule-associated protein Nucleolar and spindle associated protein 1 (NUSAP1) prevents its ubiquitination and degradation; stabilized NUSAP1 then upregulates LDHA, forming a NUSAP1-LDHA feed-forward loop that promotes glycolysis (51). Ultimately, these events may create a self-reinforcing positive feedback cycle: lactate accumulation promotes lactylation, and lactylation in turn enhances lactate production, sustaining the Lactylation-Immunosuppression Network.

However, this pathway has an intrinsic “brake.” Accumulated lactate promotes platelet-type phosphofructokinase (PFKP) lactylation (52), thereby inhibiting glycolytic activity and preventing metabolic toxicity. This could offer a potential therapeutic target. A possible strategy is to disrupt this feedback loop by blocking PFKP lactylation, rather than lactate production, further pushing cancer metabolism past its limit, and driving cells into a fatal glycolytic overload and metabolic crisis.

Lactylation can also support malignant traits. The lactylation of eukaryotic translation elongation factor 1A2 (eEF1A2) was reported to boost protein synthesis and promote tumor growth (53). Similarly, lactylation of SRY-box transcription factor 9 increases stemness, migration, and invasion (54). Furthermore, this cycle may confer resistance in tumor cells with resistance by activating autophagy. H3K18la epigenetically upregulates autophagy facilitators, such as the Rubicon-like autophagy enhancer (*RUBCNL*), complementing a direct lactylation of autophagy kinases such as Vacuolar protein sorting 34, which enhances their activity (55, 56). Enhanced autophagy can improve resistance to cytotoxic drugs such as cisplatin (28) and potentially contribute to an immunosuppressive TME with high lactate levels.

## 4 Targeting the network: emerging therapeutic strategies

The Lactylation-Immunosuppression Network offers many therapeutic targets. Approaches can be grouped into three types: cut the lactate supply, hit the enzymatic machinery, or combine methods.

### 4.1 Cutting off the fuel - targeting lactate production and transport

LDHA inhibitors—oxamate, gossypol, FX11—block the conversion of pyruvate-to-lactate, thereby reducing intratumoral lactate levels (31, 57, 58). This strategy is shown to resensitize chemoresistant tumors. It may remodel the TME by alleviating T cell suppression and synergize with Programmed cell death protein 1 (PD-1) blockade (59). Systemic toxicity remains a risk with broad metabolic blockers; therefore, targeting lactate export has emerged as a second tactic. Monocarboxylate transporter (MCT) inhibitors disrupt lactate exchange between tumor and stroma (60, 61). Notably, the selective MCT1 inhibitor AZD3965 has shown safety in Phase I trials and enhances anti-PD-1 efficacy (62). Interfering with lactate sensing in immune cells represents another innovative approach. Preclinical models showed that inhibiting the macrophage lactate sensor Olfactory Receptor 78 prevented M2 polarization, offering a novel way to remodel the immunosuppressive niche (63).

### 4.2 Disarming the writers and erasers - targeting lactylation enzymatic machinery

Targeting the enzymatic machinery presents challenges. The primary “writers” present a fundamental problem. These prominent writers, E1A binding protein p300 (p300), and CREB-binding protein (CBP) are notoriously promiscuous, posing broad off-target risks (64–67). A more precise approach targets Alanyl-tRNA synthetase (AARS1) to block its lactylation of p53, a specific interaction mediated by this lactate sensor (68). Other writer-focused strategies target K-Acetyltransferase 8, which promotes protein synthesis via eEF1A2 lactylation (53), or the poor-prognosis marker K-Acetyltransferase 2A (37). For instance, we can activate the mitochondrial delactylase Sirtuin 3 (SIRT3). A SIRT3 agonist (honokiol) combined with an NAD^+^ precursor was shown to restore mitochondrial function and cytotoxicity in natural killer (NK) cells (69). This suggests a promising path to reinvigorate immunity. Broad histone deacetylase (HDAC) inhibitors can also modulate lactylation-related pathways and improve immunotherapy outcomes (70–72), although they are not highly specific, limiting their use. A better approach may be to pharmacologically tune specific erasers (such as certain sirtuins or lysine delactylases), which could attenuate the immunosuppressive program with fewer off-target effects (73).

### 4.3 Combination therapies and innovative modalities

Synergistic combinations could help dismantle the Lactylation-Immunosuppression Network. Drug repurposing represents one tactic: combining liposomal irinotecan and epirubicin inhibited AARS1-mediated lactylation of the DNA repair factor Bloom syndrome protein, disrupting homologous recombination. This strategy has been found to be safe and gave 100% recurrence-free survival in a Phase I trial for anthracycline-resistant bladder cancer (74). Other novel technologies, such as the Proteolysis Targeting Chimera-based ACAT2 degrader, AP1, could disrupt key feedback loops. By degrading ACAT2, a critical node in a pancreatic cancer loop, AP1 disrupts a suppressive “lactate-cholesterol axis,” reverses M2 macrophage polarization, and enhances anti-PD-1 efficacy, increasing survival by 68% in preclinical models (49). Immunotherapy combinations can also directly remodel the immune interface. The addition of trifluoperazine to anti-PD-1 therapy suppressed the lactylation- associated factor, the transcriptional regulator Nuclear protein 1, thereby reducing both PD-L1 expression and M2 polarization. This action increased CD8^+^ T cell infiltration 2.1-fold in hepatocellular carcinoma models, bolstering checkpoint blockade efficacy (11). These interventions often converge on reinvigorating anti-tumor immunity (unleashing T cells/NK cells or re-educating TAMs), making lactylation a promising immunometabolic target.

## 5 Discussion

Though the Lactylation-Immunosuppression Network is a robust framework for understanding tumor immunosuppression, this field is still in its infancy. The key challenges must now be overcome to forge a more unifying model that guides future research toward new therapies. We therefore begin by stating what we mean by a functional node, then discuss specificity, evidence gaps, context dependence, and practical tools.

### 5.1 Defining the standard: minimal experimental criteria for network nodes

To make this field more rigorous, we use the term “node” to refer to a defined lactylation event. It should be reliably detected in the relevant context and linked to immunosuppression in a manner that can be tested. We have proposed four verification criteria:

Existence: It must be reliably detected by quantitative mass spectrometry analysis or by orthogonal validation for this lactylation.

Substrate dependence: The lactylation level can be modulated by modulating lactate/LDH activity regulation or by metabolic manipulation.

Mechanism consistency: Regulation is achieved by known regulatory factors (such as p300/CBP) or inhibitory factors.

Functional relevance: Changing the mark tracks with immunosuppressive phenotypes such as M2-like polarization. Direct perturbation evidence is preferred when available.

These criteria set a baseline. The next challenge is specificity: when does lactylation reflect function rather than background labeling?

### 5.2 The specificity dilemma: disentangling lactylation’s true function

The enzymatic machinery of the Lactylation-Immunosuppression Network is non-specific: its foremost “writers” (p300/CBP) and “erasers” (HDACs, Sirtuins) also control acetylation (64–67). Because the same enzymes target multiple modifications, it is difficult to attribute the influence of a general inhibitor to the prevention of lactylation or other acylations. Most current interventions are therefore incapable of isolating the contributions of lactylation.

The identification of “readers” remains similarly elusive. Whereas the chromatin protein Chromobox protein homolog 3 has been suggested as a reader (39), its specificity for lactyl-lysine has not been fully validated. This absence of distinctive writers and readers complicates attributing of specific functional outcomes to lactylation versus other modifications. We are left with a key question: is lactylation a primary driver of tumor immunosuppression, or merely a byproduct of different signaling pathways? To identify lactylation as a dominant factor, it is necessary to develop more selective biochemical and genetic tools. This specificity problem is compounded by where the field has looked most. Many mechanistic links still center on a few histone marks, especially H3K18la.

### 5.3 Beyond H3K18la: charting the pan-lactylome

The initial focus on H3K18la was a significant limitation. It limits our vision to histones and ignores a broader field. Proteomics research shows that this view is not complete. Lysine lactylation is widespread, affecting thousands of non-histone proteins (75), suggesting that its influence must far exceed histone-mediated gene regulation (75).

This bias is pronounced in T cell research. For CD8^+^ T cells, research has mainly focused on how histone lactylation (e.g., H3K9la and H3K18la) regulates mitochondrial function and cell fate (24, 30). But the non-histone lactylation networks in these same cells remain almost entirely unexplored. In contrast, specific examples have emerged in Tregs. MOESIN lactylation, for instance, reinforces immunosuppression by enhancing the TGF-β pathway (33). These findings kludge a fragmented and incomplete picture. A comprehensive, unbiased map of the “pan-lactylome” across all immune cell types is awaited. We shall map this entire network using advanced proteomics and functional assays to understand how this modification controls immunosuppression fully.

Non-histone lactylation is more frequently observed in Tregs than in CD8^+^ T cells. Biology and detection bias likely both contribute. In the TME, Tregs efficiently use lactate, so lactate-linked protein changes are more readily observed. CD8^+^ T cells in an acidic, nutrient-poor TME may show only faint lactylation signals, which are easy to overlook. Moreover, Histones are abundant and tractable, while non-histone lactylation is sparse and distributed. We therefore highlight subset-resolved lactylome profiling strategies in Section 5.5. The same lactate-rich milieu does not yield a single outcome, suggesting that context and thresholds as key determinants of when lactylation becomes functionally visible.

### 5.4 The “double-edged sword” of lactylation and the “kinetic threshold” model

Lactate and lactylation play a contradictory role in the TME. Moderate lactate accumulation during early immune surveillance can help the anti-tumor immune response by enhancing the stemness of CD8^+^ T cells (76). But this beneficial state disappears in the advanced tumor. High concentrations of lactate (10–30 mM) in advanced tumors are associated with immunosuppression and can facilitate immune escape (77, 78). Complicating matters, this initial immune-promoting effect is more consistent with acetylation rather than lactylation (76). This discovery raises an important question: Is lactylation itself directly promotes the anti-tumor response, or is its function purely inhibitory and only active under high lactate conditions?

A key distinction is that lactate-driven lactylation is not inherently malignant. In acute non-malignant inflammation, lactate can act as a delayed brake limiting glycolytic overdrive and supporting resolution. In pro-inflammatory macrophages, exogenous lactate induces pyruvate kinase M2 (PKM2) lactylation, shifting PKM2 toward its active tetramer, dampening glycolysis, and favoring a reparative state (14). Tumors are different. The Warburg effect sustains high lactate over time, erasing this temporal brake and promoting persistent histone and non-histone lactylation that can reinforce immunosuppressive programs and glycolytic feedback loops.

We propose that this context-dependent behavior is controlled by the availability of metabolic substrate, similar to the dependence of acetylation on the level of acetyl-CoA (79). Specifically, we hypothesize a “Kinetic Threshold” model: the functional impact of lactylation remains latent until the accumulation of lactate-derived lactyl-CoA exceeds a putative threshold. Several lines of evidence support this hypothesis: Although there is a special GTP-specific succinyl coenzyme A synthase that can produce acetyl-CoA (80), its cellular level is about 20–350 times lower than that of acetyl-CoA (81). In addition, p300/CBP has a much lower affinity for lactyl-CoA (82). These factors indicate that, under normal conditions, the scarcity of intranuclear lactyl-CoA limits lactylation, allowing acetylation to dominate. However, in the TME of high lactate, excessive lactate may cause intranuclear lactyl-CoA to exceed the threshold. In this way, p300/CBP can efficiently catalyze lactylation and activate strong tumor-promoting processes. This model helps to explain the contradictory role of lactate. It also shows how important it is to prove that lactylation causes these immune changes, not just to show correlation.

To distinguish causation from correlation, we propose direct tests of this model. The key is to separate cytosolic lactate from the nuclear lactyl-CoA pool. One approach is to limit nuclear lactyl-CoA while keeping cytosolic lactate high, then quantify lactyl-CoA in nuclear and cytosolic fractions by targeted metabolomics. If the model is correct, lowering nuclear lactyl-CoA should reduce p300-dependent histone lactylation such as H3K18la and weaken immunosuppressive transcriptional outputs such as ARG1. These ideas make testable predictions. Turning them into evidence will require sharper tools and cell-type-resolved measurements.

### 5.5 A technological imperative for rigorous validation

Moving from association to mechanism in lactylation research requires innovative tools. Key advances include:

High-specificity metabolic profiling: Ultrasensitive mass spectrometry to quantify the scarce nuclear lactyl-CoA pool amid abundant acetyl-CoA, enabling comprehensive mapping of the lactylome and detection of threshold-crossing events.

Real-time lactyl-CoA sensors: Genetically encoded biosensors to visualize lactyl-CoA dynamics in live cells, allowing direct testing of the kinetic threshold model within the nucleus of immune and tumor cells.

Precision epigenetic editing: CRISPR/Cas9-based strategies (e.g., single-lysine K-to-R mutations that abolish specific lactylation sites) to determine whether individual lactylation events are necessary and sufficient for immunosuppressive phenotypes.

T cell subset–resolved lactylome profiling: Purify matched Tregs and CD8^+^ T cells, enrich pan-Kla peptides, and quantify them by high-sensitivity DIA, ideally with ion mobility. Validate key sites by targeted MS and loss-of-site perturbation with functional readouts.

In summary, the Lactylation-Immunosuppression Network offers a conceptual metabolic-epigenetic axis in cancer. It integrates durable epigenetic reprogramming (histone lactylation) with rapid functional modulation (non-histone lactylation). This dual mechanism highlights numerous potential therapeutic targets and offers promises for immunometabolic therapy. However, fully exploiting this insight will depend on addressing the key challenges of enzymatic specificity and deciphering the context-dependent signaling of lactate.
